# Cross-sectional associations of personal efforts and beliefs and depressive symptoms among older adults in India

**DOI:** 10.1038/s41598-022-17578-1

**Published:** 2022-08-01

**Authors:** Priya Saravanakumar, T. Muhammad, Shobhit Srivastava

**Affiliations:** 1 School of Nursing and Midwifery, Faculty of Health, University of Technology Sydney, Building 10, Level 7, 235 Jones St, Ultimo, NSW 2007 Australia; 2 International Institute for Population Sciences, Mumbai, 400088 India

**Keywords:** Health care, Geriatrics, Health policy, Health services, Public health, Quality of life

## Abstract

Whilst there is growing evidence on the increased vulnerability of older adults to depression, there is limited research on potentially mitigative factors against symptoms of depression at a population level. This research examined associations of possible protective factors (personal efforts and beliefs) and depressive symptoms among older adults in India. This cross-sectional study used data from the Longitudinal Aging Study in India with 31,464 respondents aged 60 years and above. Depressive symptoms were assessed using the 10-item Centre for Epidemiologic Studies Depression Scale. Multivariable linear regression was used while exploring the associated factors of depressive symptoms. The mean score of depressive symptoms was 2.94 (CI 2.92, 2.96). Older adults who engaged in moderate [aCoef: −0.11, CI −0.18, −0.05], vigorous [aCoef: −0.09, CI −0.16, −0.03], or both types of physical activity [aCoef: −0.10, CI −0.19, −0.02] had lower likelihood of depressive symptoms in comparison to those who were physically inactive. Older adults who participated in social activities were less likely to have depressive symptoms [aCoef: −0.44, CI −0.50, −0.39] compared to their socially inactive counterparts. Further, older adults who perceived religion as very important [aCoef: −0.29, CI −0.41, −0.17], who had high life satisfaction [aCoef: −0.78, CI −0.82, −0.73], who had good self-perceived health [aCoef: −0.29, CI −0.33, −0.25] and those who had high self-perceived social standing [aCoef: −0.39, CI −0.47, −0.31] had lower likelihood of depressive symptoms in comparison to their respective counterparts. Physical activity, social participation, voluntary work and financial contribution to family, religiosity, life satisfaction, self-perceived health and self-perceived social standing are associated with lower likelihood of depressive symptoms among community-dwelling older adults in this study. Future longitudinal studies should explore these factors that can guide interventions against depression in old age.

## Introduction

Complex health and wellbeing needs of the rapidly growing older adult population continue to challenge the health care and social systems in both developed and developing countries. Amidst the developing countries, India shares this dynamic population demographic, having the second largest proportion of older adults in the world^1^. A major contributor to the disease burden is the incidence of late life depression in older adults that presents considerable economic and social concerns for many countries^2,3^. This continues to be a major public health concern in India, with additional country specific barriers^4,5^. Some of the barriers for mental health service provision for older adults at a macro level include large variation in population demographics between regions, fragmented health and social support services, varied distribution of support services between states, lack of public awareness on older adult mental health and a large reliance on familial support structures^6^. At a micro level, the seriousness of late- life depression is due to its prolonged trajectory and negative impacts on the individual’s quality of life. Presence of depressive symptoms in older adults if not intervened early, could result in increased morbidity from development of debilitating cognitive conditions such as dementia, worsening of other co-existing health conditions such as cardiovascular disease and fatal consequences such as suicide^7,8^.

**Figure 1:**
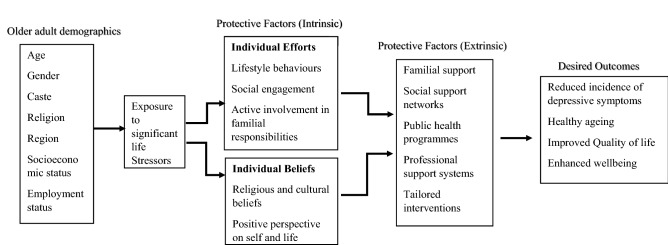
Older adults’ response to stressors—Indian context.

Adults in old age commonly encounter significant and sometimes life altering stressors such as loss of spouse, retirement, changes to familial support system and presence of more than one long-term health condition^9^. The vulnerability of older adults to depression could be explained as their response to significant stressors. How the individuals respond to stressors depends on their biophysical, psychological and socio-demographic context^10^. In addition, response to stress varies from person to person and also depends on available coping resources, both intrinsic and extrinsic^11^. In this research, we conceptualise the response of older adults to stressors in late life as influenced by an interaction between their bio-psycho-socio-demographic context and intrinsic and extrinsic coping resources for desirable positive outcomes (Fig. 1).

Intrinsic resources that have a protective effect against depression can be categorised into individual efforts and individual beliefs. Individual efforts such as pursuing an active and healthy lifestyle, being socially engaged and actively involved in familial responsibilities^12–14^ have been widely reported as contributing to healthy ageing, a sense of purpose, empowerment and quality of life^15^. Additionally, individual beliefs such as having a positive perspective on oneself and in life in general are likely to have protective effects by improving self-efficacy to cope with stress^16^. Individuals with high self- efficacy are more likely to seek social and professional support, and take initiative to combat depressive symptoms^10^. Religious and cultural beliefs are being increasingly recognised by mental health professionals as supporting the individual to have an alternative perspective on life changes due to stressors and to improve connections to promote wellbeing^17^. Extrinsic resources comprise external support systems that are available to the individual such as familial support, social support networks, public health programmes, awareness of mental health and wellbeing, and professional support system. In the Indian context, there exist additional layers of complexities such as inequitable access to above extrinsic resources for particular population cohorts such as the socially deprived, people belonging to lower caste and some regions making them more vulnerable to depression^18^. In addition, employment status, education and awareness of support programmes are frequently pointed out by researchers as factors associated with late life depression^19^.

Stressors can be potential growth opportunities, where older adults positively adapt to changes in their life, develop positive coping strategies, identify new support resources and continue to experience wellbeing. However, when stressors are multiple and overwhelming, and beyond the individual’s coping resources, they may succumb to the negative consequences such as depression. This is an important consideration for health professionals and policy makers. An individual who is struggling to cope with the stressors could be helped by professional interventions. At a macro level this also means the public health policy makers could promote protective intrinsic behaviours by increasing awareness, screening programmes to identify depressive symptoms and to execute targeted programmes for vulnerable populations.

Emerging international population-based research reveals a positive picture, describing older adults as having resilience potential and generally able to cope well with stressors and experience wellbeing in late life^20–22^. These research reports are from developed countries where there is an established and sound social support structure. However, in the Indian context, the health and social care experience of older adults is largely varied due to socioeconomic diversity, absence of a central social support system, lack of focussed outreach public health programmes for older adults and support schemes that are varied across states and regions^6,23^. In the recent decades, there is an emerging awareness of older adult health and wellbeing, and informal support programmes offered by not-for-profit organisations^24^.

Whilst there is growing evidence on the increased vulnerability of Indian older adults to depression, there is a limited research on factors that are protective and potentially mitigative against symptoms of depression at a population level in the Indian context. In this research, we examined the associations of posited protective factors (personal efforts and beliefs) and late life depressive symptoms at an individual level and related socio-demographic characteristics among older adults in India using a large nationally-representative data.

## Methods

### Data

The present cross-sectional study is based on data from the first wave of the Longitudinal Aging Study in India (LASI) collected during 2017–18^25^. LASI is a national representative longitudinal survey of middle-and older-aged Indians (i.e., aged 45 years or older) and their spouses who reside in the same households, irrespective of age. The LASI provides rich information on demographics, morbidity, health behaviour factors, and physical health of the aging population in India. The LASI survey adopted a multistage stratified area probability cluster sampling design. It is a nationally representative survey of 72,250 older adults aged 45 and above across all states and union territories of India. LASI is envisioned to be conducted every two years for the next 25 years^25^.

The LASI survey is conceptually comparable to the United States Health and Retirement Study (HRS) and other HRS-type surveys in various countries, including China (China Health and Retirement Longitudinal Survey) and England (English Longitudinal Study of Ageing). Along with its uniqueness of comparability with studies in other countries, LASI also considered features unique to India, including its institutional and cultural characteristics. LASI is conducted through a partnership of the International Institute of Population Sciences (IIPS), Harvard University, and the RAND Corporation^25^. Since we are interested in exploring the determinants of multimorbidity among the older adults, we restrict our attention to the subsample of the Indian older adults and limit our sample to respondents aged 60 or above. The present study data set contains 31,464 respondents, 16,366 women, and 15,098 men^25^.

### Variable description

#### Outcome variable

The respondents' depressive symptoms were assessed using the Centre for Epidemiologic Studies Depression Scale (CES-D). Seven negative symptoms (difficulty concentrating, feeling depressed, low energy, fear of something, feeling alone, bothered by things, and everything is an effort) and three positive symptoms (difficulty concentrating, feeling depressed, low energy, fear of something, feeling alone, bothered by things, and everything is an effort) were among the ten items (feeling happy, hopeful, and satisfied). In the week leading up to the interview, response possibilities included rarely or never (< 1 day), occasionally (1 or 2 days), often (3 or 4 days), and most or all of the time (5–7 days). Negative symptoms were scored zero in the rarely or never (< 1 day) and occasionally (1 or 2 days) categories, and one in the often (3 or 4 days) and most or all of the time (5–7 days) categories. The scale was used as continues variable for analysing the association.

#### Explanatory variables

The variables were categorized as personal effort, personal beliefs and socioeconomic & demographic attributes.

1. Socioeconomic and demographic attributes
  Socio-economic status was coded as poorest, poorer, middle, richer and richest. Socio-economic status was assessed using monthly per capita expenditure (MPCE). The MPCE is measured using household consumption data. Sets of 11 and 29 questions on the expenditures on food and non-food items, respectively, was used to canvas the sample households. Food expenditure was collected based on a reference period of seven days, and non-food expenditure was collected based on reference periods of 30 days and 365 days. Food and non-food expenditures have been standardised to the 30-day reference period. The MPCE is computed and used as the summary measure of consumption^25^. Employment was coded as never worked, currently working, not working and retired. Education was coded as not educated/ primary not completed, primary completed, secondary completed and higher & above. Awareness of welfare scheme was coded as no and yes. Age group was coded as 60–69 years, 70–79 years and 80 + years. Gender was coded as male and female.
  Caste was categorized as Scheduled Tribe, Scheduled Caste, Other Backward Class and others. The Scheduled Caste includes a group of population which is socially segregated and financially/economically by their low status as per Hindu caste hierarchy. The Scheduled Castes and Scheduled Tribes are among the most disadvantaged socio-economic groups in India. The OBC are the group of people who were identified as “educationally, economically and socially backward”, and are considered low in traditional caste system. The “other” caste category is identified of having higher social status. Religion was categorized as Hindu, Muslim, Christian and Others. Place of residence was categorized as rural and urban. Region was categorized as North, Central, East, Northeast, West and South.
2. Personal effort factors
  Physical activity was coded as inactivity, only moderate, only vigorous and both moderate & vigorous^26^. Quit tobacco consumption was coded as never consumed, currently consuming and quit. Social participation was coded as no and yes. The LASI survey collected information on whether a person goes out of the house for eating, goes outdoors for relaxing, plays outdoor games or exercises, visits relatives, attends cultural events, attends religious functions and attends group meetings. Voluntary work was coded as no and yes. Looking after children and grandchildren was coded as no and yes. Financial contribution to family/others was coded as no and yes.
3. Personal belief factors
  Importance of religion was coded as not important, somewhat important and very important. Life satisfaction was coded as high, medium and low^27^. Self-perceived health was coded as good (good, very good and excellent) and poor (fair and poor)^27^. Self-perceived social standing was assessed using ladder technique and the question was “Think of the ladder with 10 stairs as representing where people stand in our society. At the top of the ladder are the people who are the best off—those who have the most money, most education, and best jobs. At the bottom are the people who are the worst off—who have the least money, least education, and the worst jobs or no jobs. The higher up you are on this ladder, the closer you are to the people at the very top and the lower you are, the closer you are to the people at the very bottom of your society”. A score of 0–10 was recoded into high (8–10) medium (4–7) and low (0–3).

### Statistical analysis

Descriptive analysis along with mean was calculated for measuring initial results. Additionally, multiple linear regression was used while exploring the association between the outcome and explanatory variables. The regression estimates were reported at 95% CI (Confidence Interval). The results were presented in the form of unadjusted and adjusted coefficients. The svyset command was used to adjust the analysis for complex survey design. The individual weights were used to make the estimates nationally representative. STATA version 14 was used for the analysis. Model-1 represents the unadjusted estimates whereas model-2 represents the adjusted estimates (adjusted for personal effort, personal beliefs, and socioeconomic and demographic attributes).

## Results

Table 1 presents the sample characteristics. A proportion of 11.3% of the sample was aged 80 years and above and 52.6% were females in the current study. A proportion of 74.4% older adults were physically inactive. Also, 34% of the respondents were current consumers of smoked/smokeless tobacco, whereas 5.8% quit the tobacco consumption. A proportion of 85.6% of older adults reported participation in social activities and 30.7% reported engagement in voluntary work. Little more than 17% looked after children/grandchildren in the household, whereas only 5.8% contributed to the household financially. On the other hand, 78.8% of older adults reported that religion is very important to them. A proportion of 45.5% of older participants had high life satisfaction, whereas 52.7% had a good self-rated health and 7% had a high self-perceived social standing.

**Table 1:** Socio-economic profile of older adults in India, 2017–18.

Variables	n	%
**Socioeconomic and demographic attributes**		
Age (mean, sd)	69.2 (7.5)	
**Age group (in years)**		
60–69	18,410	58.5
70–79	9501	30.2
80 +	3553	11.3
**Gender**		
Male	14,931	47.5
Female	16,533	52.6
**Employment**		
Never worked	8308	26.4
Currently working	9397	29.9
Not working	11,469	36.5
Retired	2282	7.3
**Education**		
Not educated/Primary not completed	21,381	68
Primary completed	3520	11.2
Secondary completed	4371	13.9
Higher and above	2191	7
**Awareness of welfare schemes**		
No	12,670	40.3
Yes	18,794	59.7
**Socioeconomic status**		
Poorest	6829	21.7
Poorer	6831	21.7
Middle	6590	21
Richer	6038	19.2
Richest	5175	16.5
**Caste**		
Scheduled Class	5949	18.9
Scheduled Tribe	2556	8.1
Other Backward Class	14,231	45.2
Others	8729	27.7
**Religion**		
Hindu	25,871	82.2
Muslim	3548	11.3
Christian	900	2.9
Others	1145	3.6
**Region**		
North	3960	12.6
Central	6593	21
East	7439	23.6
Northeast	935	3
West	5401	17.2
South	7136	22.7
**Personal effort**		
**Physical activity**		
Inactive	23,408	74.4
Only moderate	2842	9
Only vigorous	3580	11.4
Both moderate and vigorous	1634	5.2
**Quit tobacco consumption**		
Never consumed	18,957	60.3
Currently consuming	10,686	34
Quit	1820	5.8
**Social participation**		
No	4524	14.4
Yes	26,940	85.6
**Voluntary work**		
No	21,806	69.3
Yes	9658	30.7
**Looking after children/grandchildren**		
No	26,074	82.9
Yes	5390	17.1
**Financial contribution to family/others**		
No	29,639	94.2
Yes	1825	5.8
**Personal belief**		
**Importance of religion**		
Not important	673	2.2
Somewhat important	5873	19
Very important	24,346	78.8
**Life satisfaction**		
High	13,822	45.5
Medium	6796	22.4
Low	9773	32.2
**Self-perceived health**		
Good	16,582	52.7
Poor	14,882	47.3
**Self-perceived social standing**		
High	2195	7
Middle	16,142	51.3
Low	13,127	41.7
**Total**	31,464	100

sd: Standard deviation.

Table 2 presents the mean score of depressive symptoms among study population by their background characteristics. Overall, mean depressive symptom was 2.94 (CI 2.92, 2.96). Older adults who did both moderate and vigorous physical activity had on average 2.99 depressive symptoms (2.91–3.08). older adults who were currently consuming tobacco had slightly higher mean score of depressive symptoms than those who never consumed tobacco. Socially active older adults had only 2.88 symptoms on average compared to 3.28 symptoms among socially inactive older adults. Similarly, those who did voluntary work had 2.88 symptoms compared to 2.96 symptoms among those who did not do voluntary work. Mean score of depressive symptoms was high among those who perceived religion as not important (mean score: 3.70, CI 3.55–3.85), those who had low life satisfaction (mean score: 3.67, CI 3.64–3.71), those who had poor perceived health (mean score: 3.31, CI 3.28–3.34), and those who had low perceived social standing (mean score: 3.07, CI 3.03–3.10) in comparison to their respective counterparts.

**Table 2:** Mean CES-D score among older adults by background characteristics, 2017–18.

Variables	Mean	95% CI
**Socioeconomic and demographic attributes**		
**Age group (in years)**		
60–69	2.92	2.9–2.95
70–79	2.91	2.87–2.95
80+	3.07	3–3.13
**Gender**		
Male	2.82	2.79–2.85
Female	3.04	3.02–3.07
**Employment**		
Never worked	3.01	2.98–3.05
Currently working	2.89	2.85–2.92
Not working	3.00	2.97–3.04
Retired	2.53	2.47–2.6
**Education**		
Not educated/Primary not completed	3.09	3.06–3.11
Primary completed	2.74	2.68–2.79
Secondary completed	2.57	2.52–2.62
Higher and above	2.52	2.45–2.58
**Awareness of welfare schemes**		
No	2.87	2.84–2.9
Yes	2.98	2.96–3.01
**Socioeconomic status**		
Poorest	3.14	3.09–3.18
Poorer	2.92	2.88–2.96
Middle	2.87	2.82–2.91
Richer	2.88	2.84–2.93
Richest	2.85	2.8–2.9
**Caste**		
Scheduled Class	3.18	3.13–3.23
Scheduled Tribe	2.85	2.81–2.9
Other Backward Class	2.94	2.91–2.97
Others	2.79	2.76–2.83
**Religion**		
Hindu	2.97	2.95–2.99
Muslim	2.83	2.77–2.89
Christian	2.91	2.85–2.97
Others	2.52	2.44–2.61
**Region**		
North	2.96	2.91–3
Central	3.32	3.26–3.37
East	2.98	2.94–3.03
Northeast	2.50	2.45–2.55
West	2.36	2.31–2.41
South	3.02	2.98–3.06
**Personal effort**		
**Physical activity**		
Inactive	2.96	2.94–2.99
Only moderate	2.85	2.78–2.91
Only vigorous	2.83	2.77–2.89
Both moderate and vigorous	2.99	2.91–3.08
**Quit tobacco consumption**		
Never consumed	2.92	2.89–2.94
Currently consuming	2.97	2.94–3
Quit	2.95	2.87–3.04
**Social participation**		
No	3.28	3.22–3.34
Yes	2.88	2.86–2.9
**Voluntary work**		
No	2.96	2.94–2.99
Yes	2.88	2.85–2.92
**Looking after children/grandchildren**		
No	2.94	2.92–2.97
Yes	2.90	2.85–2.94
**Financial contribution to family/others**		
No	2.95	2.93–2.98
Yes	2.65	2.57–2.72
**Personal belief**		
**Importance of religion**		
Not important	3.70	3.55–3.85
Somewhat important	3.24	3.19–3.28
Very important	2.92	2.9–2.94
**Life satisfaction**		
High	2.54	2.52–2.57
Medium	3.21	3.17–3.25
Low	3.67	3.64–3.71
**Self-perceived health**		
Good	2.60	2.57–2.62
Poor	3.31	3.28–3.34
**Self-perceived social standing**		
High	2.44	2.37–2.5
Middle	2.90	2.87–2.92
Low	3.07	3.03–3.1
**Total**	2.94	2.92–2.96

CI Confidence Interval.

Table 3 provides the adjusted and unadjusted regression estimates of depressive symptoms by background characteristics. While considering the personal effort factors, the following associations were observed. Older adults who engaged in moderate [aCoef: −0.11, CI:−0.18, −0.05], vigorous [aCoef: −0.09, CI −0.16, −0.03], or both moderate and vigorous physical activity [aCoef: −0.10, CI −0.19, −0.02] had lower likelihood of depressive symptoms in comparison to those who were physically inactive. Similarly, older adults who quit tobacco consumption were less likely to have depressive symptoms [uCoef: −0.10, CI −0.18, −0.01] in comparison to those who never consumed tobacco. Older adults who participated in social activities were less likely to have depressive symptoms [aCoef: −0.44, CI −0.50, −0.39] compared to their socially inactive counterparts. Those who reported involvement in voluntary work had lower likelihood of depressive symptoms [aCoef: −0.09 CI −0.16, −0.01] compared to their counterparts with no voluntary work. Also, older adults who looked after children/grandchildren [uCoef: −0.09, CI −0.15, −0.04] or contributed to family financially [aCoef: −0.13, CI −0.20, −0.06] were likely to have depressive symptoms in comparison to those who did not look after children or did not contribute financially, respectively.

**Table 3:** Regression estimates for depression among older adults in India, 2017–18.

**Variables**	**Model-1**	**Model-2**
	**Unadjusted Coef. (95% CI)**	**Adjusted Coef. (95% CI)**
**Socioeconomic and demographic attributes**		
**Age group (in years)**		
60–69	−0.15*(−0.22, −0.09)	−0.16*(−0.22, −0.1)
70–79	−0.05(−0.12, 0.02)	−0.09*(−0.15, −0.02)
80 +	Ref	Ref
**Gender**		
Male	Ref	Ref
Female	0.2*(0.16, 0.24)	0.04(−0.01, 0.09)
**Employment**		
Never worked	Ref	Ref
Currently working	−0.22*(−0.27, −0.17)	−0.03(−0.12, 0.06)
Not working	−0.02(−0.07, 0.03)	0.01(−0.05, 0.05)
Retired	−0.56*(−0.63, −0.48)	−0.13*(−0.21, −0.04)
**Education**		
Not educated/Primary not completed	Ref	Ref
Primary completed	−0.25*(−0.31, −0.19)	−0.1*(−0.16, −0.05)
Secondary completed	−0.35*(−0.41, −0.29)	−0.08*(−0.14, −0.02)
Higher and above	−0.61*(−0.69, −0.54)	−0.23*(−0.31, −0.15)
**Awareness of welfare schemes**		
No	Ref	Ref
Yes	−0.02(−0.06, 0.02)	−0.2*(−0.24, −0.16)
**Socioeconomic status**		
Poorest	Ref	Ref
Poorer	−0.12*(−0.18, −0.06)	−0.02(−0.08, 0.03)
Middle	−0.23*(−0.29, −0.17)	−0.07*(−0.13, −0.02)
Richer	−0.22*(−0.28, −0.16)	−0.03(−0.09, 0.02)
Richest	−0.32*(−0.38, −0.26)	−0.05(−0.11, 0.02)
**Caste**		
Scheduled Class	Ref	Ref
Scheduled Tribe	−0.34*(−0.41, −0.27)	−0.08*(−0.15, −0.01)
Other Backward Class	−0.11*(−0.16, −0.05)	−0.06*(−0.11, 0)
Others	−0.28*(−0.34, −0.22)	−0.04(−0.1, 0.02)
**Religion**		
Hindu	Ref	Ref
Muslim	0.02(−0.04, 0.09)	0.01(−0.06, 0.06)
Christian	−0.31*(−0.38, −0.25)	−0.01(−0.08, 0.07)
Others	−0.5*(−0.59, −0.41)	−0.39*(−0.48, −0.3)
**Region**		
North	Ref	Ref
Central	0.45*(0.38, 0.52)	0.36*(0.29, 0.43)
East	0.13*(0.07, 0.2)	−0.03(−0.09, 0.04)
Northeast	−0.4*(−0.47, −0.32)	−0.35*(−0.43, −0.27)
West	−0.44*(−0.51, −0.37)	−0.31*(−0.38, −0.25)
South	0.25*(0.19, 0.31)	0.12*(0.06, 0.18)
**Personal effort**		
**Physical activity**		
Inactive	Ref	
Only moderate	−0.10*(−0.17, −0.03)	−0.11*(−0.18, −0.05)
Only vigorous	−0.19*(−0.25, −0.13)	−0.09*(−0.16, −0.03)
Both moderate and vigorous	−0.07(−0.16, 0.02)	−0.10*(−0.19, −0.02)
**Quit tobacco consumption**		
Never consumed	Ref	Ref
Currently consuming	0.04*(0, 0.08)	−0.03(−0.07, 0.02)
Quit	−0.1*(−0.18, −0.01)	−0.05(−0.13, 0.02)
**Social participation**		
No	Ref	Ref
Yes	−0.49*(−0.55, −0.44)	−0.44*(−0.5, −0.39)
**Voluntary work**		
No	Ref	Ref
Yes	−0.14*(−0.19, −0.1)	−0.09*(−0.16, −0.01)
**Looking after children/grandchildren**		
No	Ref	Ref
Yes	−0.09*(−0.15, −0.04)	−0.04(−0.09, 0)
**Financial contribution to family/others**		
No	Ref	Ref
Yes	−0.32*(−0.4, −0.24)	−0.13*(−0.2, −0.06)
**Personal belief**		
**Importance of religion**		
Not important	Ref	Ref
Somewhat important	−0.15*(−0.28, −0.01)	−0.14*(−0.27, −0.02)
Very important	−0.52*(−0.64, −0.39)	−0.29*(−0.41, −0.17)
**Life satisfaction**		
High	−1.02*(−1.07, −0.98)	−0.78*(−0.82, −0.73)
Medium	−0.50*(−0.55, −0.45)	−0.36*(−0.41, −0.31)
Low	Ref	Ref
**Self-perceived health**		
Good	−0.58*(−0.62, −0.55)	−0.29*(−0.33, −0.25)
Poor	Ref	Ref
**Self-perceived social standing**		
High	−0.65*(−0.73, −0.57)	−0.39*(−0.47, −0.31)
Middle	−0.21*(−0.25, −0.17)	−0.2*(−0.24, −0.16)
Low	Ref	Ref

Ref: Reference; CI: Confidence interval; *if p < 0.05; Model-1 represents the unadjusted estimates; Model-2 represent the adjusted estimates (adjusted for personal effort, personal beliefs and socioeconomic and demographic attributes).

On the other hand, considering factors which are linked to individuals’ personal belief, older adults who perceived religion as very important [aCoef: −0.29, CI −0.41, −0.17], who had high life satisfaction [aCoef: −0.78, CI −0.82, −0.73], who had good self-perceived health [aCoef: −0.29, CI −0.33, −0.25] and those who had high self-perceived social standing [aCoef: −0.39, CI −0.47, −0.31] had lower likelihood of depressive symptoms in comparison to their respective counterparts. Furthermore, older adults with higher education were less likely to have depressive symptoms [aCoef: −0.23, CI −0.31, −0.15] in comparison to those with no formal education. Also, older adults who had awareness of welfare schemes had lower likelihood of depressive symptoms [aCoef: −0.20, CI −0.24, −0.16] in comparison to those who were unaware of such schemes.

## Discussion

Older people report depressive symptoms when they are distressed or worried about the implication of their symptoms. Similarly, self-neglecting behaviours and medical comorbidities were reported higher among older depressed persons^28^. A previous study found that about 60% to 70% of depressed patients had at least one, while 30% to 40% had two or more, somatic or psychiatric comorbidities, and had an impaired response and remission rate during treatment^29^. Depression occurring in older patients is often undetected or inadequately treated^30^, and this is particularly common in older age groups because older old persons may show less mood and motivational symptoms compared with younger old persons^31^. On the other hand, other health-related problems and disability may also lead older people to mention several symptoms, which makes screening instruments necessary to identify those who are under stress or depressed^32,33^. The current study using the CES-D scale of screening for depressive symptoms, explored its possible protective factors (intrinsic as well as extrinsic) among older Indian adults. The current findings confirmed the hypothesis that personal efforts and beliefs have significant negative association with depressive symptoms among older adults.

Several follow-up and cross-sectional studies have documented that engagement in leisure and non-leisure time physical activity is associated with lower risk of depressive symptoms among older adults^34–38^. However, some studies found that only higher intensity activity is associated with improved mental wellbeing^39^. The findings of our study show that older persons who engage in either moderate or vigorous or both types of physical activities were less likely to suffer from depressive symptoms. The findings need to be confirmed with future prospective cohort studies or intervention trials.

Age-related losses, such as loss of family members and friends, increase as people age, leading to increased feelings of loneliness and mental illnesses^40–42^, whereas, a large number of longitudinal studies suggest that participation in social activities offers a promising mechanism to bolster resilience and protects against depressive symptoms among older adults^43–45^. The particular importance of social participation in ameliorating the mental health of older individuals which is evident in some Indian studies^46–48^, is also consistent with our study. Moreover, social engagements may be especially protective for older Indian adults, given the importance of family ties and social connectedness in particular cultural contexts^49^. Similarly, voluntary work in older adults was another protective factor against depressive symptoms in our study which is in line with previous studies that have demonstrated the potential of volunteering in reducing depressive symptoms for older adults^50–52^.

Another noteworthy finding of the current study was that older adults’ contribution to household functioning in ways of looking after child/grandchild and financial contribution predicted lower depressive symptoms. The possible explanation again centers on the particular importance of societal norms of respect for senior members and connectedness among family ties^27,53^. Family in Indian populations is considered foundational and integral for their care and support and thus, the protective effects of such support, and its potential to reduce depressive symptoms in older adults, should be investigated further.

Importantly, the current study found religiosity as a predictor against depressive symptoms. The finding is consistent with the larger body of research documenting a protective effect of religiosity and spirituality in regard to depressive symptoms^54–56^. However, as documented, lack of faith as part of hopelessness may be another symptom that characterizes clinical depression^57^. The association between religiosity and depressive symptoms warrants further exploration using cohort designs, as it might hold potential to inform care approaches in treating Indian older adults with depression, and it is out of scope for the current study. In addition to this, high life satisfaction, good self-perceived health and social status were significantly associated with lower risk of depressive symptoms in this study, and these findings correspond to previous studies showing statistically significant associations between perceived health and wellbeing and depressive disorders^58–61^.

In this research, it was found that higher levels of education, being retired from work and awareness of social welfare schemes were negatively related to depressive symptoms among older adults. These findings suggest that several socioeconomic variables may play major roles in reducing depressive symptoms, and the possible coping strategies among higher socioeconomic groups need to be further investigated to inform interventions as all the coping strategies may not work well or be of benefit to the wellbeing of socioeconomically poor population. The overall implication of the findings is that socioeconomic characteristics of the aged population in India play significant roles in their risk for depression and these significant factors should be taken into consideration to deliver effective mental health prevention interventions that would promote healthy ageing.

### Implications for practice

There is the need for paying attention to the unmet mental health needs of older Indian population as ageing in the country comes with its associated challenges. The current findings have important implications for government, health-practitioners and individuals engaged in social work practice. Given the higher prevalence of depressive symptoms among community-dwelling older adults in India^62^, efforts are called for to reduce the risk of depressive symptoms which may occur as part of increased exposure to the age-related life stressors, by enhancing the possible protective factors including personal efforts and beliefs.

In particular, among impoverished depressive people, whose life pressures can be severe, encouraging appropriate involvement in physical, social and spiritual activities or incorporating factors of personal beliefs into a therapeutic regimen may have a benefit^63,64^. Furthermore, findings of this research indicate that bolstering support networks and encouraging active involvement in the household and filial relationships through financial contribution to family and looking after children may prevent depressive symptoms among senior members of the family^65–68^. Thus, coupling clinical services for depressive disorder with physical, social, spiritual, and family related activities is suggested. Also, in addition to formal interventions, strengthening existing social and family support networks are important areas to explore in the prevention and treatment of depression among older Indian adults. Similarly, those who report that religion is important in their lives, could be encouraged to participate in related activities, which could aid in coping when facing further age-related stressful events and ameliorate depressive symptoms.

Apart from this, the following strategies could be followed as part of enhancing the personal beliefs and in turn, overall wellbeing of older adults. Firstly, we recommend government initiatives such as increasing older adults’ active engagement through incentivising and acknowledging volunteering work, increasing income through old age pension, providing subsidised health insurance cover to ameliorate their overall welfare. These can be crucial preliminary steps in bettering their socioeconomic status and decreasing depression risk and improving their quality of life^69,70^. Secondly, an effective multidisciplinary network of health services could be established for screening of older persons for mental illnesses enabling timely access to mental health services, and these services should be extended to rural areas where the depression prevalence is higher in comparison to urban areas^71^. Thirdly, health-promotional activities such as promoting awareness, and increasing opportunities to engage in physical and social activities would aid in enhancing their personal beliefs, increase life satisfaction and improve their overall quality of life^27,72,73^. However, additional research is required to explore effective approaches to implement the above mentioned strategies into practice among diverse older Indian cohorts.

### Strengths and limitations

In this study, we examined both personal efforts and personal beliefs as separate aspects of the individuals’ intrinsic resources to potentially mitigate depressive symptoms. A major strength of this study is the large sample size of the study participants, providing information on older adults aged 60 years and above residing in rural and urban areas of the country and the inclusion of a wide range of potentially confounding variables. The study had a cross-sectional design, not allowing for causality measurements and hence, we did not focus on causal pathways. Furthermore, the self-report of several variables such as depressive symptoms, tobacco use and importance of religion are subject to measurement errors and limited to the measures that were part of the survey. Further, information on stressful life events associated with late-life depression including death of relatives, friends or caregivers and other socioeconomic stress factors like deterioration of financial status^74^, was not collected and should be included in future studies. Similarly, other potential factors related to late-life depression (demographic and clinical) such as prior history of depression, psychotherapy, psychiatric medication/psychiatric disorders; current psychiatric medication; social support; and comorbidities were not included in the current analyses. Although social participation and self-perceived health partly covered some of them, future studies using multiple waves of the LASI data, should investigate the longitudinal associations of protective factors of late-life depression by including all these factors. Finally, respondents who were institutionalised were not included in the survey, and thus we may have underestimated the prevalence of depressive symptoms in the current study.

## Conclusion

Personal efforts such as physical activity, social participation, voluntary work and financial contribution to family and personal beliefs such as religiosity, higher life satisfaction, good self-perceived health and a higher self-perceived social standing are identified to be associated with lesser likelihood of depressive symptoms in older persons. Future longitudinal studies should focus on determining the associations of these factors with depression in old age that can be used to guide interventions. These potentially protective factors may also help in formulating public health initiatives to reduce the risk of depression and improve quality of life among older adults in the Indian context.

### Ethics approval and consent to participate

The survey agencies that conducted the field survey for the data collection have collected prior informed consent (written and verbal) from all the participants. The Indian Council of Medical Research (ICMR) extended the necessary guidance and ethical approval for conducting the LASI survey.

All methods related to the current analysis were carried out in accordance with relevant guidelines and regulations by the Indian Council of Medical Research (ICMR).

## Data Availability

The study uses secondary data which is available in the public repository of International Institute for Population Sciences, Mumbai, accessible through https://www.iipsindia.ac.in/content/lasi-wave-i.
